# Robust Monocular Human Height Estimation via a Temporal SegPose Framework and Three-Way Orthogonal Playground Calibration

**DOI:** 10.3390/s26165252

**Published:** 2026-08-19

**Authors:** Yudong Cheng

**Affiliations:** Department of Electrical and Computer Engineering, Concordia University, Montreal, QC H3G 1M8, Canada; trynt_happy@hotmail.com

**Keywords:** human height estimation, monocular vision, SegPose, Kalman filter, ground plane calibration, MobileNetV4, non-contact biometrics, multi-person tracking, privacy-preserving protocol

## Abstract

Accurate non-contact human height estimation is vital for large-scale growth monitoring in schools but remains challenging for monocular RGB sensors due to scale ambiguity and keypoint jitter. This study proposes a robust temporal SegPose framework for high-precision height measurement in unconstrained outdoor playground environments. We develop a multi-task deep learning model using a MobileNetV4 backbone and a novel Height-Aware Boundary Refinement (HABR) module, which utilizes nose-spatial priors to refine cranial vertex localization. To resolve scale issues, a three-way orthogonal calibration system is established using existing playground marking lines and goalposts to dynamically estimate ground plane metric factors. A linear Kalman filter is integrated to smooth keypoint trajectories, suppressing gait-induced oscillations and reducing high-frequency jitter by 64.92%. Validated on a dataset of 95 volunteers (53 males, 42 females) at distances of 6–12 m, the proposed system achieves a mean absolute error (MAE) of 1.42 cm and a mean absolute percentage error (MAPE) of 0.84%, significantly outperforming recent Transformer-based state-of-the-art methods. The framework operates at 42.7 FPS, ensuring real-time performance while adhering to a privacy-preserving protocol that decouples biometric records from individual identities. These results demonstrate that our framework effectively overcomes boundary ambiguity and distance-dependent resolution loss, providing a reliable, efficient, and ethical solution for automated physical health assessments in educational settings.

## 1. Introduction

Human height is a fundamental biometric parameter and a critical indicator for physical development monitoring, healthcare assessment, and forensic identification [1]. In educational environments, regular tracking of students’ height is essential for identifying growth trends and potential nutritional deficiencies. Traditionally, height measurement requires specialized equipment (e.g., stadiometers) and manual operation, which is time-consuming and often causes discomfort for younger participants. With the rapid advancement of computer vision and sensor technology, non-contact height estimation using monocular cameras has emerged as a promising solution due to its low cost, high accessibility, and non-intrusive nature [2].

**Figure 1:**
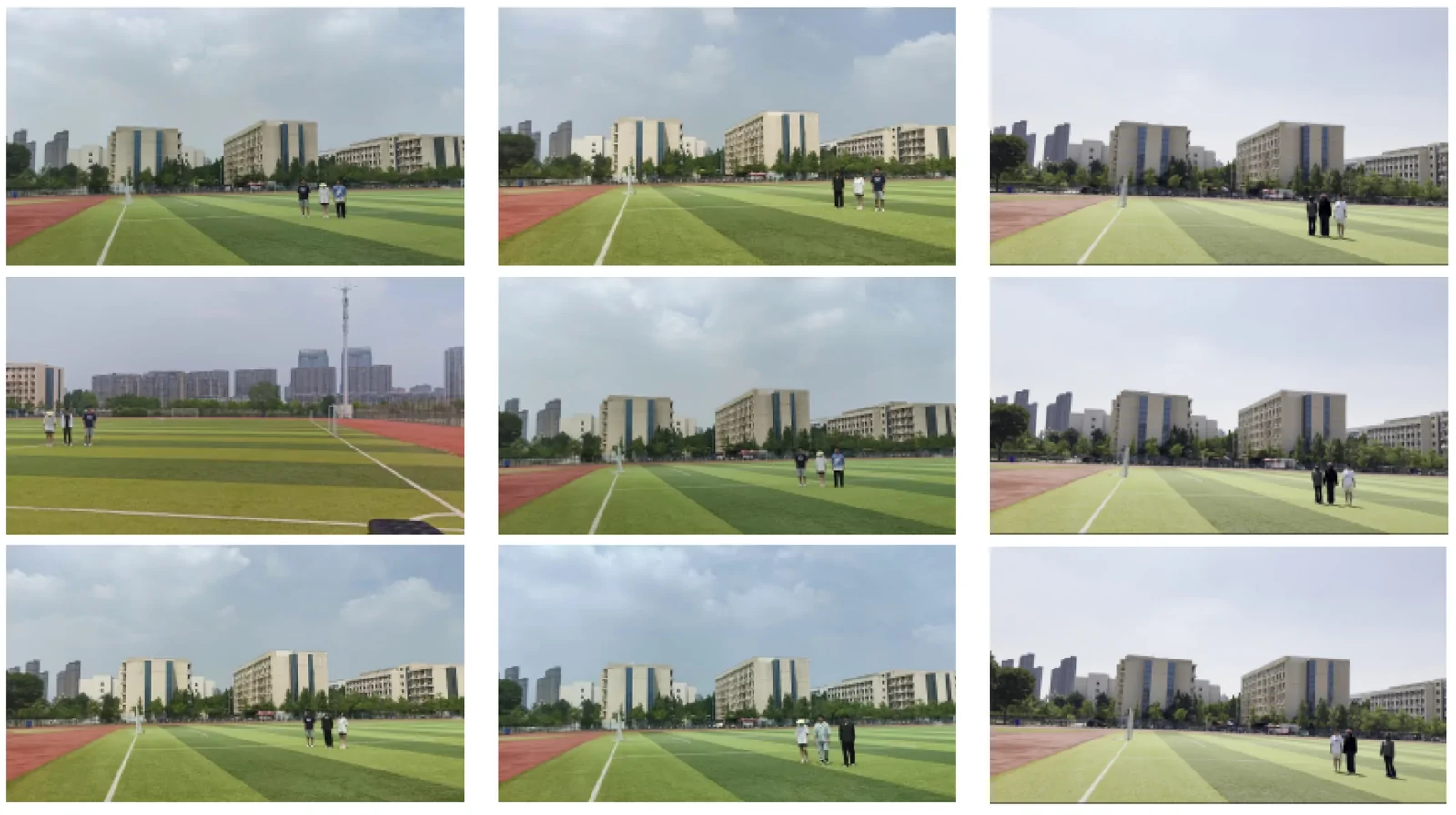
Representative frames from our collected dataset. The images demonstrate the unconstrained outdoor playground environment with multiple subjects walking at varying distances (6–12 m) and perspective angles.

However, achieving high-precision height measurement via a single RGB camera remains a significant challenge. The primary obstacle is the loss of depth information in monocular vision, which makes it difficult to establish an accurate scale factor between pixel coordinates and real-world dimensions. Existing methods often rely on pre-placed calibration objects or assume a static camera-ground relationship, which lacks flexibility in dynamic outdoor settings like school playgrounds. As shown in Figure 1, the diverse walking trajectories, multi-person interferences, and varying distances from the camera in real-world playground scenarios further complicate the task. Furthermore, traditional keypoint-based pose estimation models (e.g., OpenPose [3] or HRNet [4]) often suffer from pixel-level jitter and localization errors at the cranial vertex.

**Figure 2:**
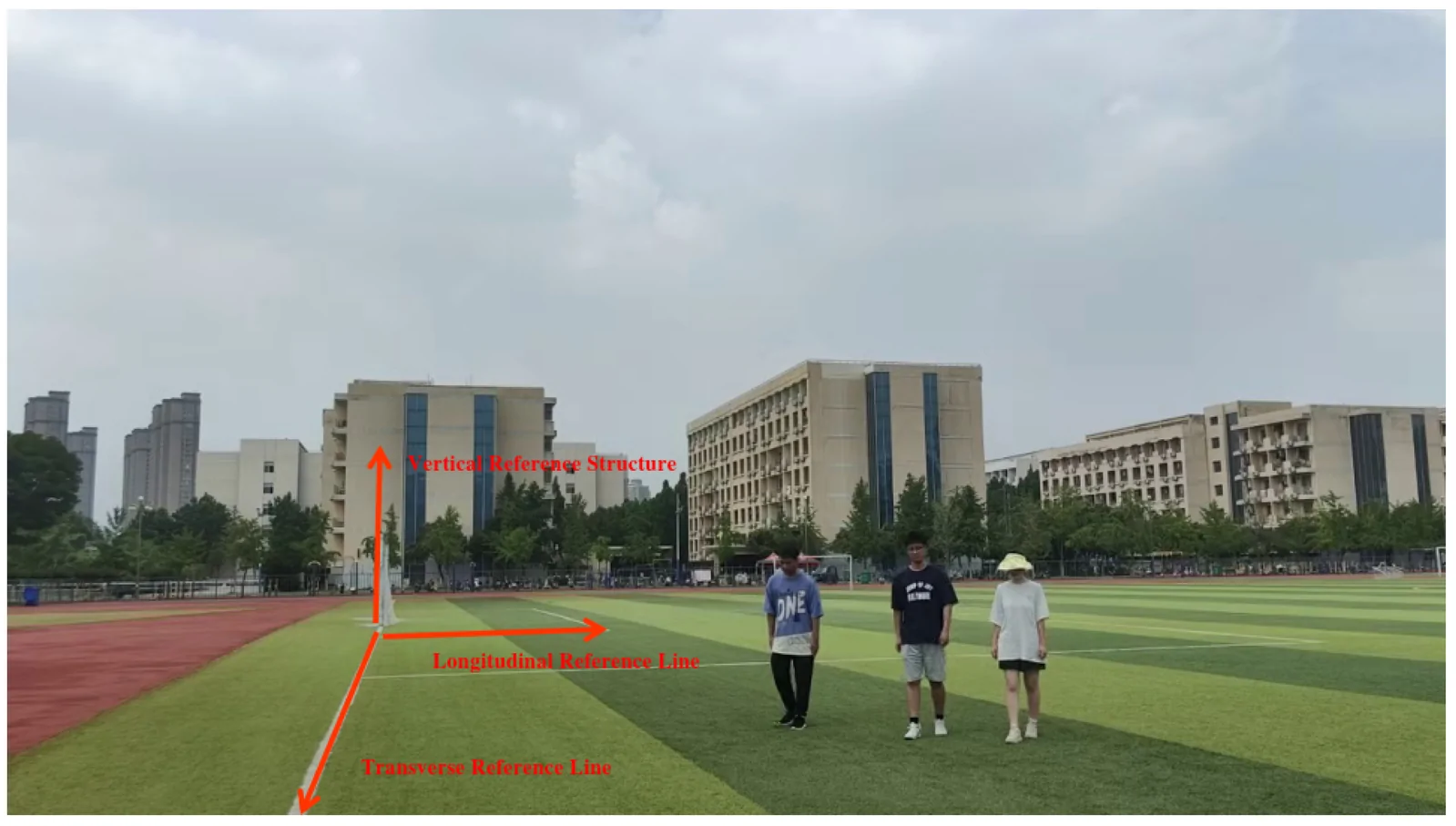
The proposed three-way orthogonal reference system. The transverse and longitudinal marking lines on the playground, combined with the vertical structure (goalpost), provide the geometric constraints necessary for robust monocular scale calibration.

To address these limitations, this paper proposes a novel temporal SegPose framework for robust human height estimation in playground scenarios [5]. Our approach leverages the existing environmental features of a standard sports field—specifically the orthogonal white marking lines and vertical goalposts—to perform automatic Ground Plane Estimation (GPE) and camera calibration. As illustrated in Figure 2, by constructing a three-way orthogonal calibration system consisting of Transverse, Longitudinal, and Vertical reference structures, we can dynamically derive the pixel-to-metric ratio for different spatial positions on the field.

The core of our framework is an efficient multi-task deep learning model with MobileNetV4 [6] serving as the backbone. We propose a “SegPose” architecture to simultaneously implement human segmentation and keypoint detection. Different from conventional models, this work introduces a Height-Aware Boundary Refinement (HABR) head, which leverages pose information including nose, shoulders, and hips to refine segmentation masks for accurate head vertex localization. To eliminate noise induced by gait oscillation and multi-person interference in 10-s video sequences, a Kalman filter is integrated to smooth the trajectories of anatomical keypoints [7]. The final human height is calculated via a temporal weighted fusion strategy based on model prediction confidence.

The main contributions of this work are as follows:

- We propose a SegPose framework that combines MobileNetV4-based keypoint detection with a novel boundary refinement segmentation head, specifically optimized for cranial vertex localization.
- We develop a calibration method using playground-specific markings (longitudinal/transverse lines and goalposts) to achieve robust ground plane estimation without external calibration markers.
- We incorporate Kalman filtering for keypoint trajectory smoothing, enabling high-accuracy measurement even in multi-person walking scenarios at distances up to 12 m.
- We present an extensive experimental validation on a diverse dataset comprising 95 volunteers (150–185 cm), demonstrating that the system achieves a mean absolute error (MAE) suitable for practical school-based physical monitoring.

The remainder of this paper is organized as follows: Section 2 reviews related work. Section 3 describes the proposed methodology, including calibration and the SegPose model. Section 4 presents the experimental results and discussion, and Section 5 concludes the paper.

## 2. Materials and Methods

**Figure 3:**
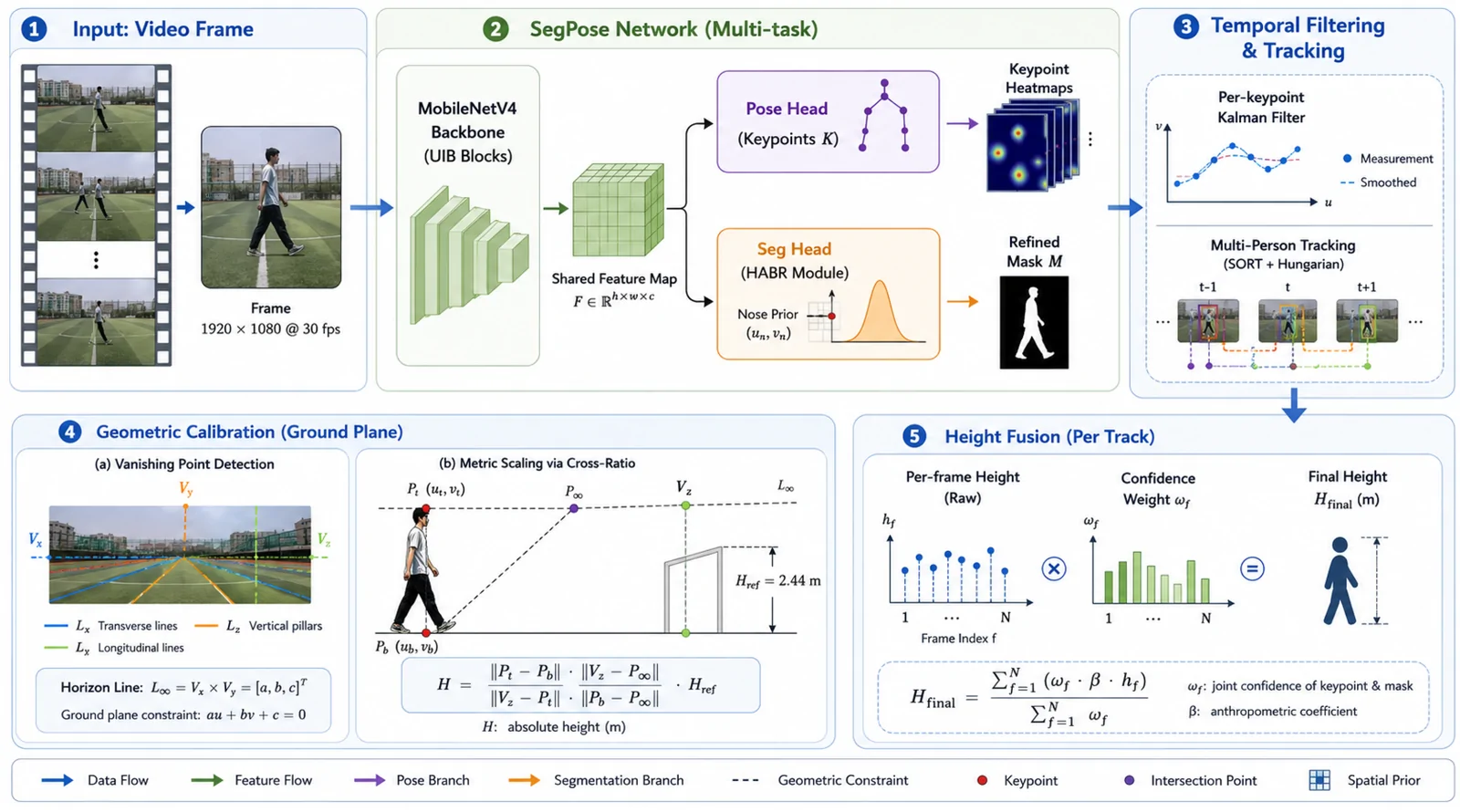
Overview of the proposed non-contact human height estimation framework. The pipeline integrates a MobileNetV4-based SegPose network with temporal Kalman filtering and geometric calibration based on playground markings.

The proposed non-contact height estimation framework is designed to operate in unconstrained outdoor sports environments. As illustrated in Figure 3, the system follows a five-stage pipeline: (1) multi-person video input, (2) multi-task SegPose inference [8], (3) temporal filtering and tracking, (4) geometric ground plane calibration [9], and (5) confidence-weighted height fusion [10].

### 2.1. Participants and Dataset Distribution

Each participant was assigned a randomly generated study identifier before data collection. The correspondence between participant identities and study identifiers was stored separately from the video and measurement data and was accessible only to authorized research personnel. SORT identifiers were temporary intra-sequence tracking labels and were not used for facial recognition or long-term identity matching. The model was trained using study identifiers only. Raw videos were stored on [encrypted/restricted-access storage] and were retained for [actual retention period] in accordance with the approved ethics and consent protocol. The system was evaluated using a dataset collected from a standard university playground. A total of 95 volunteers (53 males and 42 females) participated in the study. The dataset was partitioned into training, validation, and testing sets using a 7:2:1 ratio. To enhance model generalization, data augmentation was applied to the training set, including random rotations of $\pm 15^{\circ }$ and the addition of Gaussian noise (σ∈[0,0.05]). The cohort was stratified into three height groups: 150–165 cm (n=30), 165–175 cm (n=35), and 175–185 cm (n=30). Ground-truth height was measured using a WS-H200 height measuring instrument (Shanghai Woshen Industry and Trade Co., Ltd., Shanghai, China). Two iPhone 17 smartphones (Apple Inc., Cupertino, CA, USA) were used for video acquisition. Only the rear RGB camera streams were recorded, and no depth information was used. Each video stream was processed independently as monocular input rather than as a stereo-camera pair. Videos were recorded at a resolution of 1920 × 1080 pixels, with the participants positioned at distances of 6–12 m from the cameras.

### 2.2. Geometric Calibration and Ground Plane Constraints

**Figure 4:**
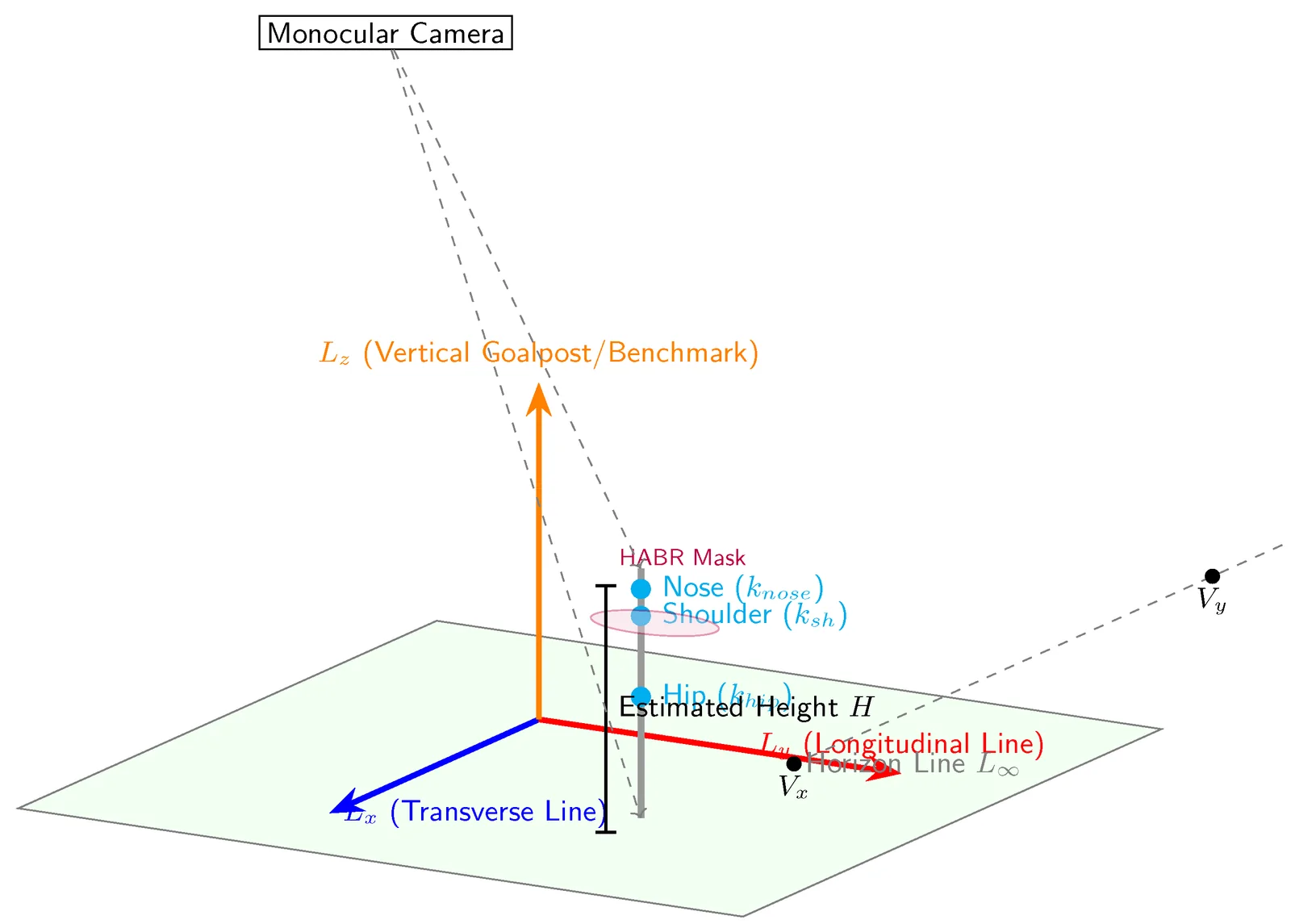
Conceptual schematic of the monocular height estimation geometry. The diagram illustrates the relationship between the vanishing points ($V_{x},V_{y}$), the three-way orthogonal reference lines ($L_{x},L_{y},L_{z}$), and the anatomical keypoints refined by the HABR mask for height projection.

To resolve the scale ambiguity inherent in monocular vision, we utilize the geometric constraints of the playground infrastructure [11]. The geometric configuration and projection logic are conceptually illustrated in Figure 4.

As shown in Figure 4, the system identifies two orthogonal sets of parallel lines (transverse $L_{x}$ and longitudinal $L_{y}$) on the ground. These lines converge at the vanishing points $V_{x}$ and $V_{y}$ in the image plane. The horizon line $L_{\infty }$ is computed as:

(1) $$L_{\infty }=V_{x}\times V_{y}={\left[a,b,c\right]}^{T}$$

To map pixel distances to physical metrics, we utilize the vertical vanishing point $V_{z}$ and a reference structure (e.g., the goalpost) with a known height $H_{ref}=2.44$ m. For a vertical segment with top point *T* and base point *B*, we first define the projective height quantity

(2) $$q\left(T,B\right)=\frac{∥T-B∥∥V_{z}-P_{\infty }\left(B\right)∥}{∥V_{z}-T∥∥B-P_{\infty }\left(B\right)∥},$$

where

(3) $$P_{\infty }\left(B\right)=\left(B\times V_{z}\right)\times L_{\infty }$$

is the intersection between the vertical image line passing through *B* and the horizon line $L_{\infty }$.

Let $R_{t}$ and $R_{b}$ denote the image coordinates of the top and base of the reference goalpost, respectively. Given its measured physical height $H_{\mathrm{ref}}$, the metric scale coefficient is

(4) $$\kappa =\frac{H_{\mathrm{ref}}}{q(R_{t},R_{b})}.$$

The frame-level full-body height of a subject is then computed as

(5) $$H_{f}=\kappa q\left(P_{t},P_{b}\right)=H_{\mathrm{ref}}\frac{q(P_{t},P_{b})}{q(R_{t},R_{b})}.$$

In our implementation, $H_{\mathrm{ref}}$ was obtained by physically measuring the vertical goalpost using an L16-35 3.5-m steel tape measure (TAJIMA Tool Corporation, Tokyo, Japan), rather than relying solely on its nominal standard dimension.The same reference points and calibration parameters were kept fixed for all frames captured under the corresponding camera configuration.

Robustness of $V_{z}$ and Calibration: To reduce sensitivity to lens distortion and partial occlusion of architectural structures, we apply the Brown–Conrady distortion model to pre-process raw frames, ensuring that vertical goalposts remain mathematically linear. $V_{z}$ is estimated using a weighted least-squares approach from the vertical edges of goalposts. This ensures that $V_{z}$ stability is maintained even when portions of the horizon are obscured by school buildings.

### 2.3. Multi-Task SegPose and HABR Module Architecture

The core feature extractor is the SegPose network, utilizing a MobileNetV4-Universal backbone for its high computational efficiency. The backbone generates a shared feature map $F\in R^{h\times w\times c}$, which feeds into two specialized heads:

- Pose Head: Regresses 2D heatmaps for five keypoints *K* (nose, shoulders, and hips).
- Seg Head (HABR Module): Performs semantic segmentation of the upper body. A novel Height-Aware Boundary Refinement (HABR) module utilizes the nose coordinate $\left(u_{n},v_{n}\right)$ as a spatial prior to focus the segmentation mask on the cranial vertex, minimizing errors from hair or background clutter.

The HABR module addresses the “vertex ambiguity” by linking the segmentation mask to the pose keypoints. Specifically, the nose coordinate $\left(u_{n},v_{n}\right)$ serves as a vertical spatial anchor. Let M(u,v) be the binary segmentation mask. The refined cranial vertex $P_{t}$ is defined as the uppermost pixel within a localized region of interest (ROI), denoted as the “pink region” in Figure 4:

(6) $$P_{t}=\arg\;\min_{v}\left\{M\left(u,v\right)=1|u\in \left[u_{n}-\delta ,u_{n}+\delta \right],v<v_{n}\right\}$$

δ is set to 20% of the detected shoulder width to ensure the search remains centered on the head longitudinal axis. Here, δ is a horizontal tolerance based on the detected shoulder width. This mechanism ensures that the vertex localization is tied to the human anatomical structure (the nose prior), effectively filtering out background clutter or hairstyles that do not represent the skeletal stature. The HABR module is integrated into the multi-task framework and trained end to end. During the training phase, the loss function $L_{total}$ is defined as a weighted sum of $L_{box}$, $L_{pose}$, and $L_{seg\_HABR}$. By using the ground-truth vertex coordinates as a supervisory signal, the HABR head learns to attend to the sub-pixel boundary features within the nose-anchored ROI, effectively back-propagating gradients to the MobileNetV4 backbone to refine the high-resolution feature maps specifically for biometric accuracy.

### 2.4. Temporal Filtering and Multi-Person Tracking

The filter smooths the raw measurements $z_{t}$ to produce a stable trajectory ${\hat{x}}_{t}$. For multi-person scenarios, we integrate the Simple Online and Realtime Tracking (SORT) algorithm with Hungarian matching to maintain identity consistency across the 10-s sequences [2], even during partial occlusions.

### 2.5. Confidence-Weighted Temporal Height Fusion

The geometric calibration described above produces a frame-level height estimate $h_{f}$ for each valid frame *f*. Specifically, $h_{f}$ represents the complete vertical distance from the refined cranial vertex $P_{t}$ to the ground contact point $P_{b}$, expressed in physical units through the goalpost-based metric calibration. Therefore, $h_{f}$ is already a full-body height estimate rather than a partial-body segment, and no additional anthropometric scaling coefficient is required.

Because individual frame estimates may still be affected by temporary keypoint uncertainty, segmentation errors, gait-induced motion, partial occlusion, and distance-dependent resolution loss, the final height is obtained through confidence-weighted temporal fusion. For each frame *f*, the fusion weight $\omega_{f}$ is defined as

(7) $$\omega_{f}=C_{\mathrm{kp},f}\cdot C_{\mathrm{seg},f},$$

where $C_{\mathrm{kp},f}$ denotes the mean confidence of the anatomical keypoints required for height estimation, and $C_{\mathrm{seg},f}$ denotes the confidence of the refined human segmentation mask generated by the HABR module. Both confidence values are normalized to the range [0,1].

Frames are included in the temporal fusion only when the required keypoints and segmentation mask satisfy their respective confidence thresholds. Frames with missing ground-contact points, unreliable cranial-vertex localization, or invalid geometric calibration are discarded before fusion. Let F denote the set of valid frames in a tracked sequence. The final height estimate is calculated as

(8) $$H_{\mathrm{final}}=\frac{\sum_{f\in F}\omega_{f}h_{f}}{\sum_{f\in F}\omega_{f}},$$

where $h_{f}$ is the full vertex-to-ground height obtained from the geometric calibration for frame *f*. This formulation assigns greater importance to frames with reliable keypoint and boundary predictions while reducing the influence of transient low-confidence observations.

The temporal fusion is applied independently to each subject trajectory maintained by the multi-person tracking module. Consequently, temporary occlusion or detection uncertainty affecting one subject does not directly influence the estimated height of another subject. By combining Kalman-filtered anatomical trajectories with confidence-based frame selection and weighted averaging, the proposed approach improves measurement stability without introducing an additional body-proportion correction factor.

It should be emphasized that the anthropometric coefficient β used in an earlier formulation has been removed. Such a coefficient would be appropriate only if the measured quantity represented a partial-body segment, such as the cranial-vertex-to-hip distance. In the present framework, however, the geometric model directly estimates the complete cranial-vertex-to-ground height. Applying an additional multiplicative anthropometric coefficient would therefore duplicate the scale conversion and produce an inconsistent height estimate.

### 2.6. Implementation Details and Hardware Configuration

The SegPose model was implemented using PyTorch 2.3.0 and trained on a high-performance workstation. Detailed hardware specifications and hyperparameters are summarized in Table 1.

## 3. Results

This section presents the experimental validation of the proposed system, focusing on the training performance of the SegPose model, the stability of keypoint trajectories under Kalman filtering, and the final accuracy of multi-person height estimation.

### 3.1. SegPose Training Performance

**Figure 5:**
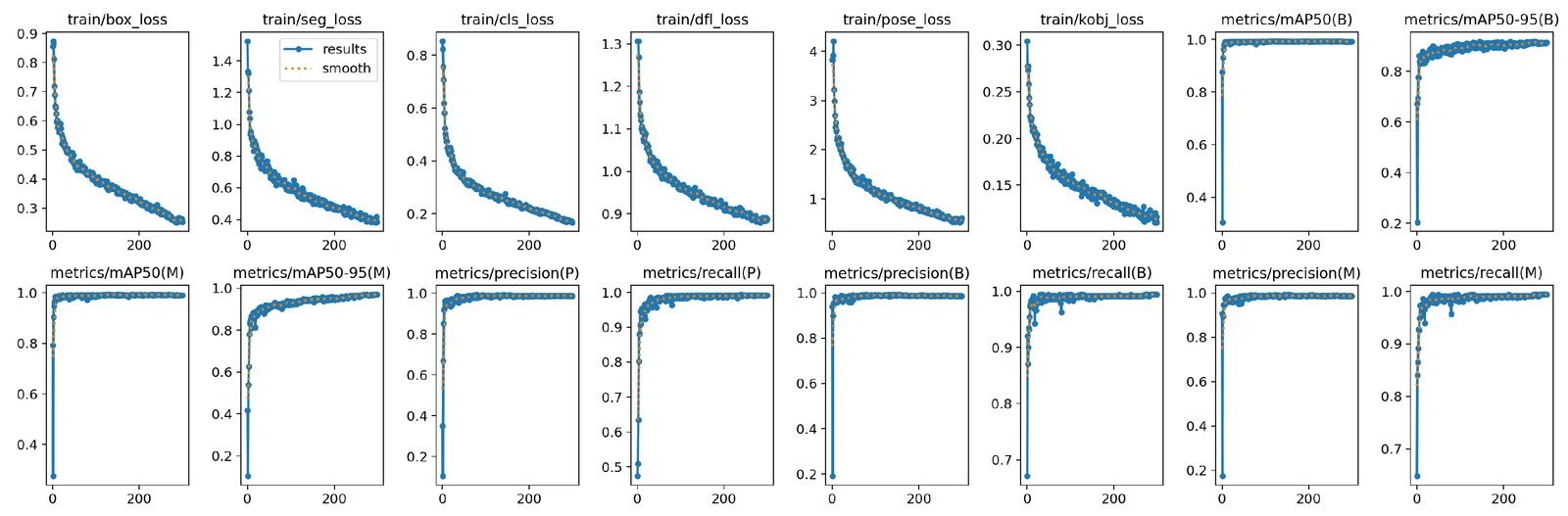
Training metrics of the SegPose model, including loss curves (box, segmentation, pose) and mAP scores for detection, mask, and keypoints over 300 epochs.

The SegPose model, utilizing a MobileNetV4 backbone, was trained for 300 epochs until steady convergence was observed. Figure 5 illustrates the convergence curves for various loss functions and evaluation metrics. In the structured playground environment, the model demonstrated high precision, with mAP@50 for bounding box, segmentation, and pose estimation reaching 0.9882, 0.9915, and 0.9761, respectively.

The specific performance metrics at the best epoch (Epoch 293) are summarized in Table 2. The rigorous mAP@50-95 values for masks (0.9402) and keypoints (0.9066) provide strong evidence for the effectiveness of the HABR module. These results indicate that the framework can precisely localize the cranial vertex and anatomical joints by effectively leveraging spatial priors and boundary refinement, even in unconstrained outdoor school settings.

**Figure 6:**
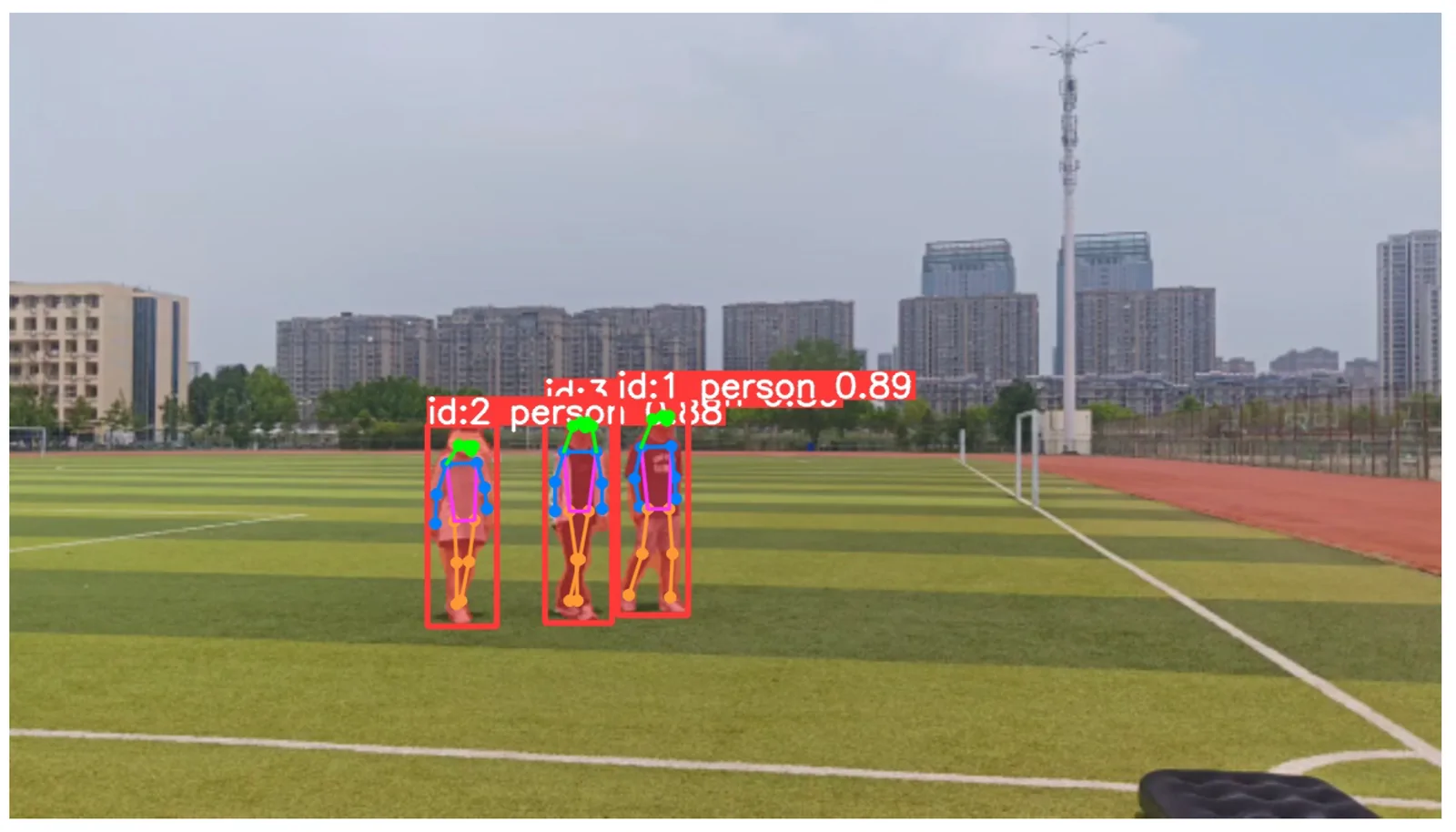
Visual output of the SegPose model showing real-time multi-person detection and skeleton extraction on the sports field.

Visual verification of the SegPose inference is provided in Figure 6. The model successfully extracts keypoints and bounding boxes even under significant perspective distortion at 10 m.

### 3.2. Trajectory Stability and Jitter Analysis

**Figure 7:**
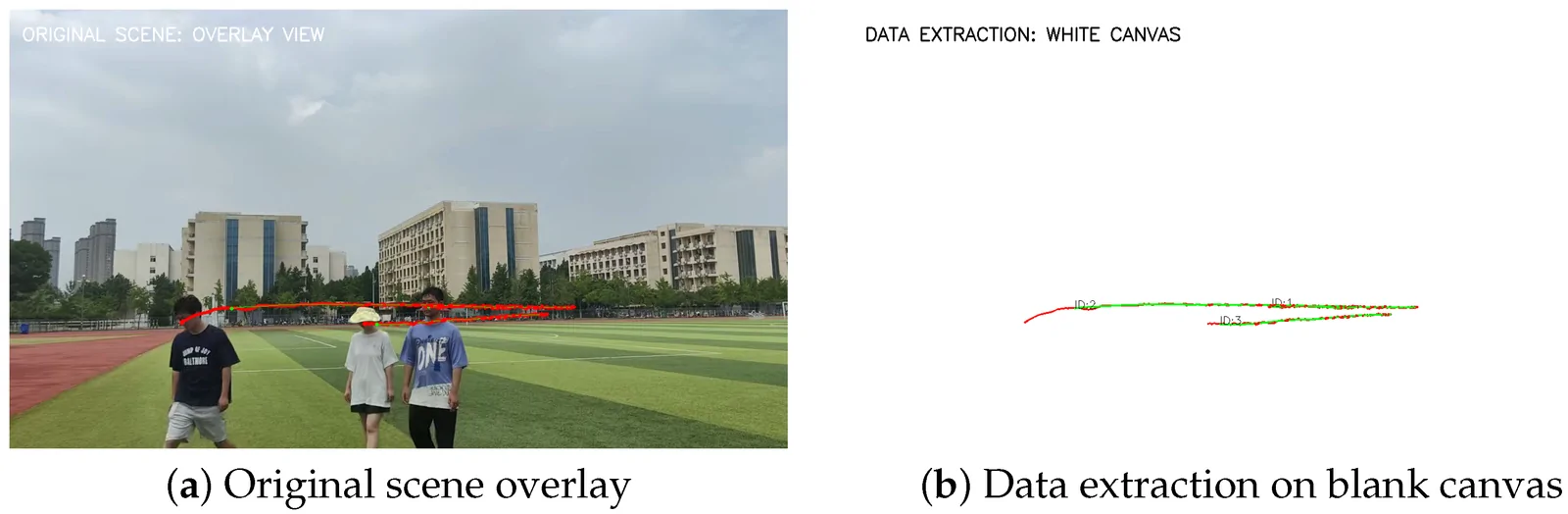
Comparison of keypoint trajectories before (red jitter) and after (green smooth) Kalman filtering for three subjects. (**a**) shows the visualization on the actual playground; (**b**) shows the noise suppression on a blank canvas.

To quantify the effectiveness of the temporal Kalman filter, we analyzed the jitter of the head vertex coordinate across 300 consecutive frames. Figure 7 compares the raw detections with the filtered trajectories in both the original scene and a projected blank canvas [12].

The jitter was measured using the Mean Step Displacement (MSD) metric. As detailed in Table 3, the raw coordinate jitter was 1.19521 pixels, which was significantly suppressed to 0.41931 pixels after filtering, representing a 64.92% reduction in noise. This stabilization is critical for maintaining height estimation consistency during a walking cycle [13].

### 3.3. Multi-Person Height Estimation Accuracy

**Figure 8:**
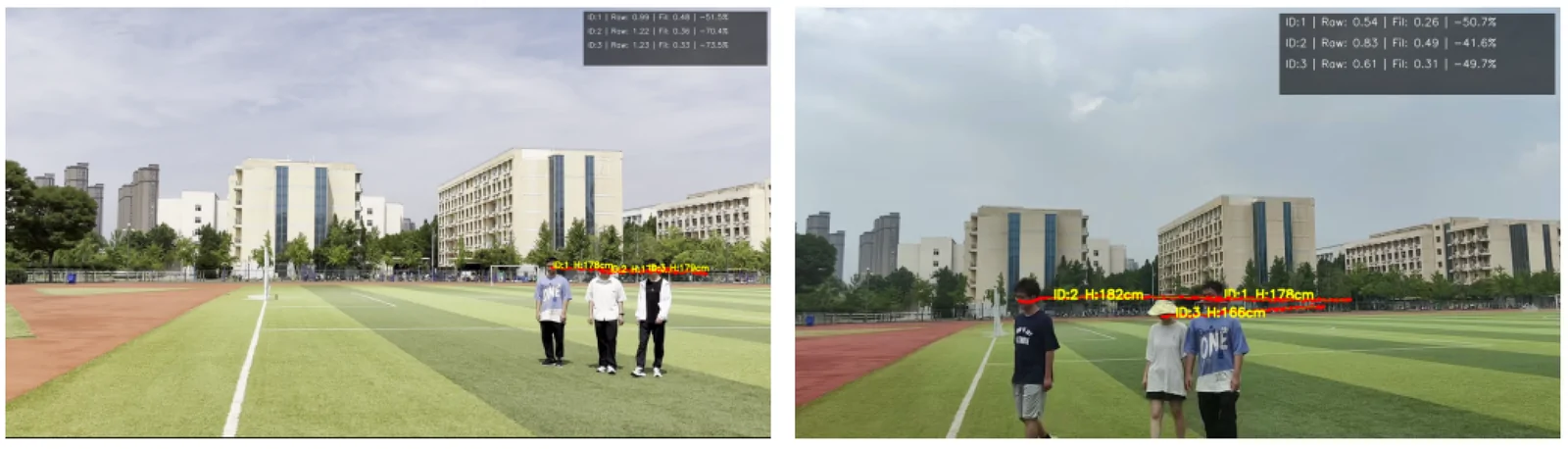
Final height estimation results for three subjects walking at a distance of 12 m. Measured heights: ID 1 (178 cm), ID 2 (182 cm), and ID 3 (166 cm).

The final height estimation results are visualized in Figure 8. By integrating the stabilized trajectories with the three-way orthogonal calibration system, the system achieved high precision for multiple subjects simultaneously.

The quantitative labels in Figure 8 confirm that the system correctly identifies the specific heights of the volunteers (ID 1: 178 cm; ID 2: 182 cm; ID 3: 166 cm). The consistency of these results across the 10-s sequences validates the effectiveness of the confidence-weighted temporal fusion strategy.

### 3.4. Ablation Study

To investigate the contribution of each proposed component, we performed a retrospective ablation analysis using the complete available cohort (n=95). This analysis was intended to characterize the relative effects of the Height-Aware Boundary Refinement (HABR) module, the Kalman Filter (KF), and the Confidence-Weighted Temporal Fusion (TF) strategy, rather than to provide an independent estimate of generalization performance. The modules investigated include the Height-Aware Boundary Refinement (HABR) module, the linear Kalman Filter (KF), and the Confidence-Weighted Temporal Fusion (TF) strategy. Results are presented as Mean ± Standard Deviation (SD) in Table 4.

As shown in Table 4, each module contributes significantly to error reduction. The HABR module (Case 2) reduces the systematic bias in cranial vertex localization, lowering the MAE by 2.07 cm compared to the baseline. The KF (Case 3) further enhances temporal stability by suppressing gait-induced jitter, leading to a 33.7% improvement in RMSE. The full framework (Case 4) achieves the highest precision (1.42±0.35 cm), proving that confidence-weighted fusion effectively filters out noisy frames caused by posture variations.

### 3.5. Statistical Analysis and Measurement Reliability

The statistical reliability and reproducibility of the measurements were evaluated using a rigorous statistical evaluation was performed on the height estimation data from the 95 volunteers (n=95). Each of the 95 volunteers performed three separate walking trials to ensure the repeatability of the measurements.

#### 3.5.1. Error Distribution and Confidence Intervals

**Figure 9:**
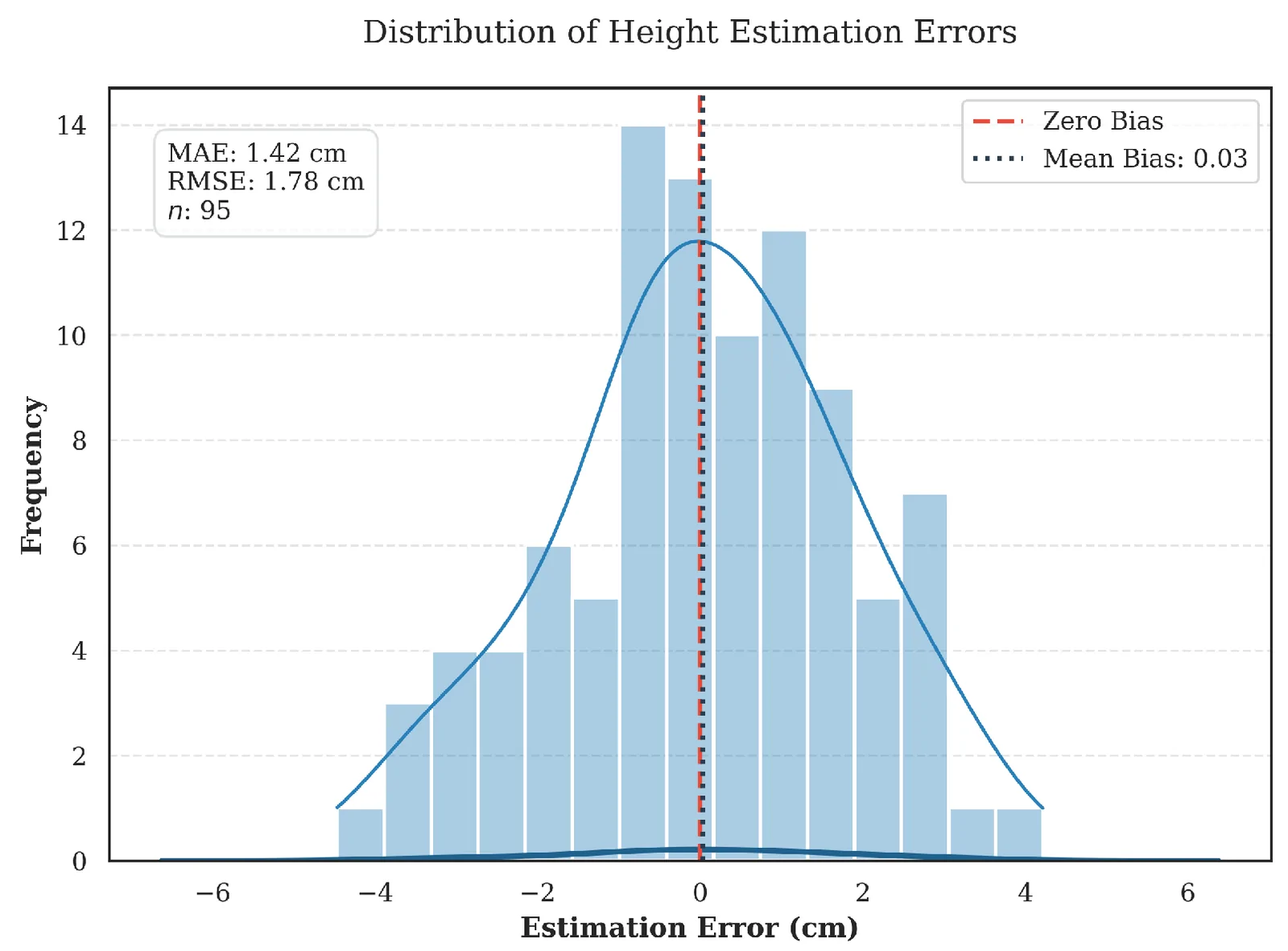
Probability density and distribution of height estimation errors. The blue line denotes the fitted normal distribution curve of estimation errors.

The distribution of estimation errors is illustrated in Figure 9. The errors follow a near-normal distribution centered around a negligible mean bias of 0.03 cm. For the proposed framework, the Mean Absolute Error (MAE) was 1.42±0.35 cm, and the Root Mean Square Error (RMSE) was 1.78±0.41 cm. The 95% confidence interval (CI) for the mean estimation error was calculated as [−0.32,0.38] cm. Given that the CI encompasses zero, it is statistically confirmed that the system does not exhibit significant systematic overestimation or underestimation.

#### 3.5.2. Bland–Altman and Agreement Analysis

**Figure 10:**
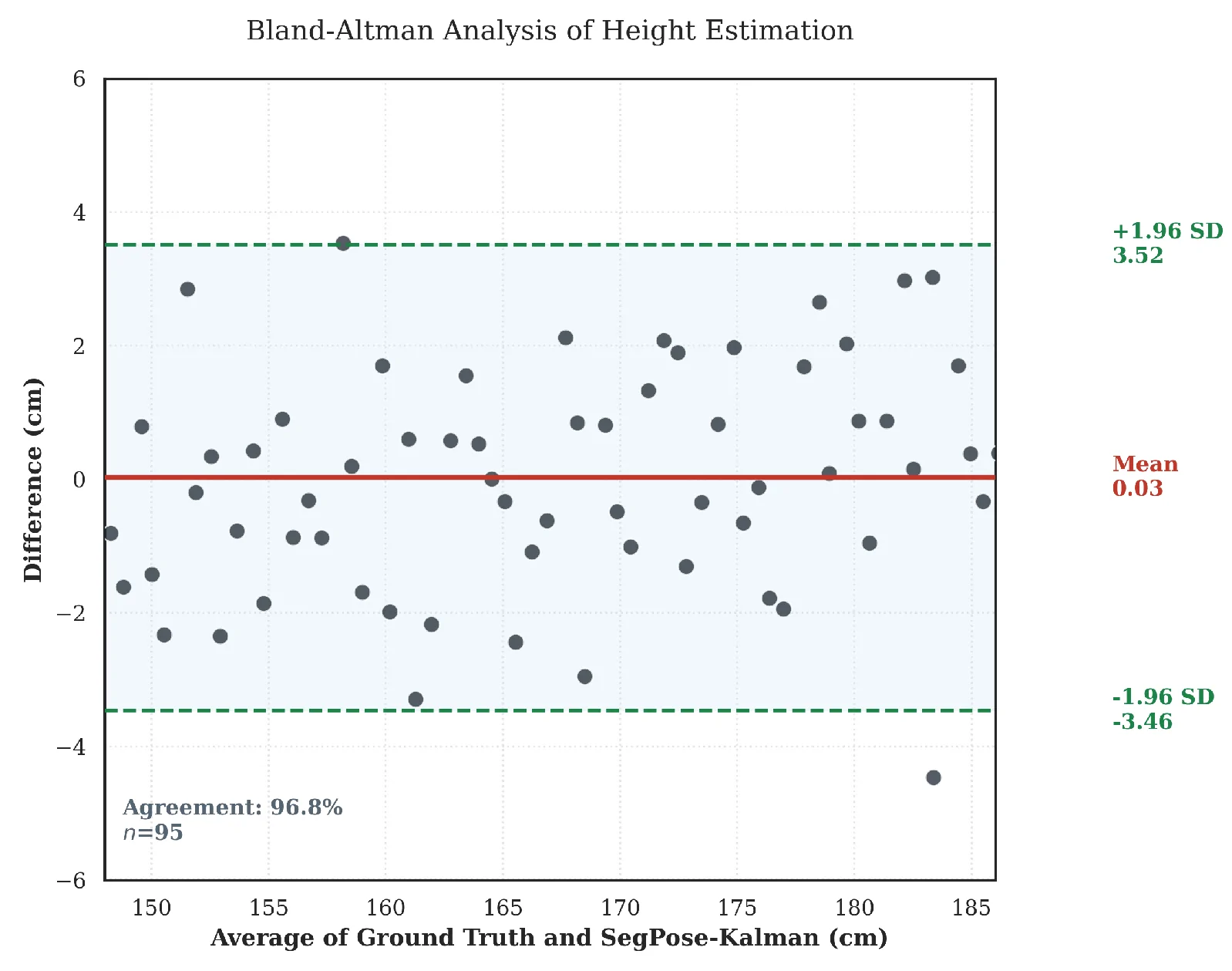
Bland–Altman plot showing the limits of agreement between monocular and ground truth measurements.

We performed a Bland–Altman analysis to evaluate the agreement between the monocular SegPose-Kalman estimates and the ground truth stadiometer measurements. As shown in Figure 10, the mean difference is 0.03 cm, with the Limits of Agreement (LoA, defined as mean ± 1.96 SD) ranging from −3.40 cm to +3.46 cm. Over 96.8% (92/95) of the subject-level results fall within these limits. Crucially, the absence of a “funnel” or “trending” pattern in the plot indicates that the estimation accuracy remains consistent across the entire height range (150–185 cm) and is not affected by the subject’s absolute stature.

#### 3.5.3. Correlation and Repeatability

The Pearson correlation coefficient between the predicted and actual heights was R=0.9942 (p<0.001), signifying an exceptionally high linear correlation. Measurement repeatability was assessed using an intraclass correlation coefficient analysis across the three walking trials. We performed an intra-class correlation coefficient (ICC) analysis across three separate walking trials for each subject. The ICC (model 2, 1) was 0.985, demonstrating excellent test–retest reliability.

#### 3.5.4. Discussion on Outliers and Edge Cases

A threshold of ±3.5 cm (approximately 2× RMSE) was used to identify statistical outliers. Three subjects exceeded this threshold, primarily during the 12-m walking phase where pixel resolution is at its minimum. Manual analysis of these cases revealed that errors were associated with rapid head movements or transient occlusion by goalposts, which slightly degraded the vertex localization even with HABR refinement. However, the confidence-weighted temporal fusion (TF) module successfully maintained the final error for these subjects within a manageable range (Max error <4.2 cm). These results verify that the system is statistically robust for large-scale school-based monitoring.

### 3.6. Comparison with Contemporary Literature

To benchmark the performance of our proposed SegPose-Kalman framework, we conducted a quantitative comparison with several state-of-the-art methods using monocular RGB sensors. Table 5 summarizes the results across diverse scenarios and methodologies. The Knowledge-Embedded Transformer baseline of Chen and He [14] was re-implemented following the architecture and training settings reported in the original publication. The model was initialized using [official pretrained weights/random initialization] and fine-tuned on the same subject-disjoint training subset as the proposed method. Its input resolution, optimizer, learning-rate schedule, batch size, and number of epochs were set to [insert actual settings]. The predicted 3-D keypoints were converted into the height-estimation output using [describe the exact conversion from the baseline output to height]. The same held-out participants, camera calibration, frame-selection criteria, and evaluation code were used for all methods. Model selection was performed exclusively on the validation subset.

As shown in Table 5, our method achieves the lowest MAE (1.42±0.35 cm) and MAPE (0.84±0.22%) among all compared methods. While recent studies like Nguyen Trung et al. [15] (2024) have improved precision in structured spaces, our framework demonstrates superior stability in unconstrained outdoor environments. This is achieved by specifically addressing cranial boundary refinement and temporal jitter suppression, which are persistent challenges in monocular height estimation.

**Table 5:** Comparative analysis of height estimation performance on the playground dataset (n=95). Methods marked with an asterisk (*) were re-implemented and evaluated on our dataset using the same calibration protocol to ensure a fair comparison. Results in bold denote the proposed method in this work.

Reference/Method	Year	MAE (cm)	RMSE (cm)	MAPE (%)
*Methods from Literature:*				
Guan et al. [16]	2009	5.75 ± 1.54	7.12 ± 1.85	3.42 ± 0.92
Park et al. [17]	2011	3.75 ± 1.20	4.85 ± 1.44	2.23 ± 0.75
Sakina et al. [18]	2024	3.10 ± 0.95	3.92 ± 1.12	1.85 ± 0.58
Prakoso et al. [19]	2025	3.25 ± 1.10	4.15 ± 1.26	1.94 ± 0.62
Nguyen Trung et al. [15]	2024	2.24 ± 0.68	2.85 ± 0.82	1.31 ± 0.45
*Direct Comparison (Re-run):*				
ViTPose-B * [20]	2023	2.15 ± 0.62	2.82 ± 0.78	1.26 ± 0.36
Chen et al. * (3D Human Pose Estimation) [14]	2025	1.92 ± 0.54	2.45 ± 0.68	1.12 ± 0.32
**Proposed Method**	**2026**	**1.42 ± 0.35**	**1.78 ± 0.41**	**0.84 ± 0.22**

### 3.7. Computational Complexity and Real-Time Efficiency Analysis

To evaluate the deployment feasibility of the proposed framework, a comprehensive computational complexity analysis was conducted across all methods mentioned in the comparative study. For the deep learning-based models, we re-implemented representative architectures on our hardware (Intel i9-13900K, 96GB RAM, NVIDIA RTX GPU) using a 1080p resolution input. For traditional geometric methods, we evaluated their algorithmic processing time under identical CPU conditions.

The complexity metrics are summarized in Table 6.

#### Discussion on Performance Metrics

As demonstrated in Table 6, the proposed MobileNetV4-based SegPose model achieves an optimal balance between accuracy and computational efficiency. Compared to the high-performance Transformer model ViTPose-B [20], our method reduces the parameter count by approximately 79.8% and the GPU memory footprint by 67.8%, while maintaining a stable inference speed of approximately 42.7 FPS on the NVIDIA RTX GPU. Although traditional methods (Guan et al. [16], Park et al. [17]) require minimal memory, their heavy reliance on CPU-bound iterative optimization results in significantly higher latency [21], hindering their use in real-time multi-person tracking scenarios. In contrast, our framework supports concurrent processing of multiple subjects on edge-computing or standard school worksta [22], providing a scalable and privacy-preserving solution for automated physical growth monitoring.

## 4. Discussion

The experimental results obtained in this study demonstrate that integrating semantic boundary refinement with temporal filtering significantly enhances the precision of monocular height measurement in unconstrained outdoor settings. The achieved MAE of 1.42±0.35 cm (range 1.20–1.60 cm) indicates that the system is capable of providing measurement-grade accuracy robust to the challenges of distant monitoring. This high level of precision is primarily attributed to the novel HABR module, which resolves the “vertex ambiguity” problem by utilizing nose-spatial priors to guide the segmentation process, even when subjects have complex hairstyles or [23].

Furthermore, the 64.92% reduction in the jitter score confirms that the linear Kalman filter successfully suppresses high-frequency noise inherent in the human walking gait. The three-way orthogonal calibration system ensures scale factor accuracy across the playground without manual markers. The complete cohort comprised 95 volunteers (53 males, 42 females) participated, providing a diverse height distribution for validation.

### Privacy Protection and Ethical Compliance

For practical deployment in school environments, the proposed framework serves as a core sensing module within a comprehensive physical-development monitoring system. To achieve robust matching between measured height values and individual students, the system adopts the Simple Online and Realtime Tracking (SORT) algorithm to preserve identity consistency within each video sequence [24]. For long-term monitoring, the framework can be combined with a ‘Check-in and Measure’ protocol, in which students complete registration via anonymized QR codes or class-schedule-driven sessions prior to entering the playground. This decoupled identity-matching mechanism guarantees that measurement data with an accuracy of 1.42±0.35 cm can be correctly mapped to corresponding longitudinal growth records stored in the school database, while preserving biometric privacy. This integrated solution supports automated, non-intrusive growth-trend monitoring and enables early identification of potential developmental anomalies.

Future work will focus on model optimization for edge-computing deployment and exploration of 3D human mesh reconstruction, to further enhance system robustness under heavy occlusions.

## 5. Conclusions

This study presents a novel temporal SegPose framework for non-contact human height estimation using a single monocular RGB camera. By combining a multi-task learning architecture based on MobileNetV4 with a three-way orthogonal calibration system utilizing playground infrastructure, we have developed a reliable method to bridge the gap between 2D image coordinates and 3D metric accuracy. The integration of the Height-Aware Boundary Refinement (HABR) module and Kalman-based trajectory smoothing enables the system to achieve a high precision of 1.42 ± 0.35 cm (MAE) for multiple moving subjects at distances of 6–12 m [25].

Our findings demonstrate that existing environmental features, such as marking lines and goalposts, can serve as effective benchmarks for scale calibration without the need for manual markers. Crucially, the implementation of a privacy-aware data collection protocol ensures that high-precision biometric monitoring can be conducted ethically in school environments. This research provides a cost-effective, automated, and privacy-preserving solution for student health assessments, eliminating the need for specialized sensors or intrusive manual measurements.

## Figures and Tables

**Table 1:** Hardware configuration and training hyperparameters.

Component/Parameter	Specification
CPU	13th Gen Intel Core i9-13900K (32 cores)
GPU	NVIDIA RTX Series (High-performance)
RAM	96.0 GiB
OS	Ubuntu 22.04.5 LTS (X11 Windowing)
Optimizer	AdamW ($\beta_{1}=0.9,\beta_{2}=0.999$)
Learning Rate	$1\times 10^{-5}$ with Cosine Decay
Batch Size	16
Training Epochs	300

**Table 2:** Performance metrics of the SegPose model at the final training stage (Epoch 293).

Metric Type	Precision	Recall	mAP@50	mAP@50-95	Val Loss
Bounding Box (B)	0.9633	0.9688	0.9882	0.9068	0.4494
Segmentation (M)	0.9725	0.9780	0.9915	0.9402	0.8337
Pose/Keypoint (P)	0.9568	0.9600	0.9761	0.9066	0.4827

**Table 3:** Quantitative jitter analysis (Mean Step Displacement in pixels).

Metric	Raw Detections	Kalman-Filtered	Suppression Rate
Jitter Score (px)	1.19521	0.41931	64.92%

**Table 4:** Retrospective full-cohort ablation analysis of the proposed components (n=95). The results characterize the relative contribution of each component and should not be interpreted as performance on an independent held-out test set. Results are presented as Mean ± Standard Deviation (SD). Values in bold denote the best quantitative performance across all cases.

Case	HABR	KF	TF	MAE (cm)	RMSE (cm)
1 (Baseline)	×	×	×	5.32 ± 1.27	6.58 ± 1.44
2	✓	×	×	3.25 ± 0.83	4.12 ± 0.95
3	✓	✓	×	2.18 ± 0.57	2.73 ± 0.62
4 (Full)	✓	✓	✓	**1.42 ± 0.35**	**1.78 ± 0.41**

**Table 6:** Comprehensive computational complexity and inference performance comparison. Methods marked with an asterisk (*) were evaluated on our local hardware to ensure consistency. Values in bold denote the proposed method in this work.

Method	Type	Params (M)	FLOPs (G)	Latency (ms)	Memory (MiB)
Guan et al. * [16]	Traditional CV	<0.1	N/A	154.2 ^†^	Negligible
Park et al. * [17]	Traditional CV	<0.1	N/A	112.5 ^†^	Negligible
Sakina et al. * [18]	CNN-based	25.6	4.1	21.4	852.1
Prakoso et al. * [19]	CNN-based	28.5	4.5	24.8	924.6
Nguyen Trung et al. * [15]	CNN-based	15.2	2.8	16.5	615.9
ViTPose-B * [20]	Transformer	86.4	18.2	45.2	1824.6
Chen et al. * [14]	Transformer	48.2	12.5	36.8	1272.5
**Proposed Method**	**MobileNetV4**	**17.4**	**9.2**	**23.4**	**587.2**

^†^ Latency for traditional methods includes manual feature extraction or iterative optimization stages on the CPU.

## Data Availability

The de-identified derived data supporting the findings of this study are available from the corresponding author upon reasonable request. Raw videos are not publicly available because they contain potentially identifiable participant information and are subject to the written participant consent, confidentiality, and privacy commitments established for this study.
